# Intergrader Agreement on Qualitative and Quantitative Assessment of Diabetic Retinopathy Severity Using Ultra-Widefield Imaging: INSPIRED Study Report 1

**DOI:** 10.3390/diagnostics15141831

**Published:** 2025-07-21

**Authors:** Eleonora Riotto, Wei-Shan Tsai, Hagar Khalid, Francesca Lamanna, Louise Roch, Medha Manoj, Sobha Sivaprasad

**Affiliations:** 1 Moorfields Eye Hospital NHS Foundation Trust, 162 City Road, London EC1V 2PD, UK; eleonora.riotto@nhs.net (E.R.); wei-shan.tsai@nhs.net (W.-S.T.); hagar.khalid@nhs.net (H.K.); francesca.lamanna1@nhs.net (F.L.); 2 Hampshire Hospitals NHS Foundation Trust, Aldermaston Road, Basingstoke RG24 9NA, UK; 3 Boehringer Ingelheim International GmbH, Birkendorfer Str. 65, 88397 Biberach, Germany; lcjroch@gmail.com; 4 Leicester Medical School, George Davies Centre, Lancaster Rd, Leicester LE1 7HA, UK

**Keywords:** diabetic retinopathy, intergrader agreement, retinal non-perfusion, predominant peripheral lesions, ultra-widefield fluorescein angiography

## Abstract

**Background/Objectives**: Discrepancies in diabetic retinopathy (DR) grading are well-documented, with retinal non-perfusion (RNP) quantification posing greater challenges. This study assessed intergrader agreement in DR evaluation, focusing on qualitative severity grading and quantitative RNP measurement. We aimed to improve agreement through structured consensus meetings. **Methods**: A retrospective analysis of 100 comparisons from 50 eyes (36 patients) was conducted. Two paired medical retina fellows graded ultra-widefield color fundus photographs (CFP) and fundus fluorescein angiography (FFA) images. CFP assessments included DR severity using the International Clinical Diabetic Retinopathy (ICDR) grading system, DR Severity Scale (DRSS), and predominantly peripheral lesions (PPL). FFA-based RNP was defined as capillary loss with grayscale matching the foveal avascular zone. Weekly adjudication by a senior specialist resolved discrepancies. Intergrader agreement was evaluated using Cohen’s kappa (qualitative DRSS) and intraclass correlation coefficients (ICC) (quantitative RNP). Bland–Altman analysis assessed bias and variability. **Results**: After eight consensus meetings, CFP grading agreement improved to excellent: kappa = 91% (ICDR DR severity), 89% (DRSS), and 89% (PPL). FFA-based PPL agreement reached 100%. For RNP, the non-perfusion index (NPI) showed moderate overall ICC (0.49), with regional ICCs ranging from 0.40 to 0.57 (highest in the nasal region, ICC = 0.57). Bland–Altman analysis revealed a mean NPI difference of 0.12 (limits: −0.11 to 0.35), indicating acceptable variability despite outliers. **Conclusions**: Structured consensus training achieved excellent intergrader agreement for DR severity and PPL grading, supporting the clinical reliability of ultra-widefield imaging. However, RNP measurement variability underscores the need for standardized protocols and automated tools to enhance reproducibility. This process is critical for developing robust AI-based screening systems.

## 1. Introduction

Diabetic retinopathy (DR) is a leading cause of vision impairment and blindness among working-age adults worldwide, with the progression to proliferative diabetic retinopathy (PDR) closely linked to increasing retinal ischemia [1]. Accurate assessment of DR severity, particularly in the context of ischemia and PDR, is critical for guiding treatment decisions and preventing vision-threatening complications [2]. Fundus fluorescein angiography (FFA) has long been the gold standard for evaluating retinal perfusion and ischemic changes [3]. Recently, the advent of advanced imaging technologies, such as Optos ultra-widefield fluorescein angiography (UWFA) (Optos 200Tx, Dunfermline, Scotland, UK), has expanded the ability to visualize retinal vasculature in unprecedented detail, including peripheral lesions that are often missed by traditional imaging modalities [4]. The Optos UWFA system, with its ability to capture up to 200 degrees of the retina in a single image, provides a unique opportunity to improve the accuracy and reproducibility of ischemia and PDR grading [3,5,6].

Recent studies have highlighted the significance of predominantly peripheral retinal lesions (PPLs) in DR progression [7,8,9,10,11]. PPLs are defined as DR lesions with a greater extent outside the standard ETDRS fields compared to those inside. Silva et al. demonstrated that the presence of PPLs was associated with a higher risk of DR progression in both ultra-widefield (UWF) color images and Optos UWFA, potentially due to their correlation with greater retinal non-perfusion (RNP) areas on UWFA [7]. The DRCR Protocol AA also reported that the presence of FFA PPL was associated with a greater risk of disease worsening over 4 years, independent of baseline Diabetic Retinopathy Severity Scale (DRSS) score [9].

The non-perfusion index (NPI) has emerged as a critical metric for quantifying retinal ischemia, providing a standardized approach to assess areas of capillary dropout and non-perfused retina. For example, the SCORE study group utilized a grid system featuring four concentric circles divided into nine subfields across the macula to more accurately measure the extent of capillary loss, blood, edema, and fluorescein leakage [12]. The introduction of UWF imaging led to various methods of quantification of ischemia, requiring standardized grading grids to systematically analyze its expansive retinal coverage while maintaining reliability and reproducibility [13]. A popular method is the NPI, which is the ratio of non-perfused retina to the total retinal area. This technique requires manually outlining the boundaries of both perfused and non-perfused retinal regions; however, it is time-consuming and can vary between observers. Alternatively, some studies measured non-perfusion by manually or digitally marking non-perfused zones and reported results in pixels or disc areas without accounting for the entire retinal area visualized [14,15].

Thus far, the evaluation of PPL and RNP is extremely subjective [13]. To overcome the variability of DR grading and quantitative measures of retinal ischemia, numerous studies have adopted intergrader analyses to prove the consistency of their reporting findings. Many of them have shown excellent intergrader agreement to support the reliability of their results [13,15,16,17,18,19,20,21,22]. For example, Thykjær A et al. found an overall DR grading agreement between primary and secondary graders of 93% (κ = 0.83) and a facility agreement of 96% (κ = 0.89) and 90% (κ = 0.76) for practicing ophthalmologists and hospital- based graders, respectively [16]. Midena E. et al. also identified a perfect intergrader agreement for DR grading classification (kappa = 0.998, 95% confidence interval (95%CI) = 0.997–0.999) [17]. Similarly, Wessel and colleagues demonstrated strong agreement in measuring ischemic areas using UWFA, with Pearson correlation coefficients exceeding 0.80 when manually outlining non-perfused retinal regions [14]. However, no studies have ever illustrated how they achieved this accomplishment. There is indeed a clinical need to demonstrate in what way a good consensus is reached between different clinicians so that it can be replicated by other studies and even artificial intelligence (AI). This step is crucial, as a change in RNP is considered an endpoint in the MAGIC study (NCT05681884) for evaluating new interventions to prevent progression of retinal ischemia [18].

This study aimed to assess the qualitative and quantitative intergrader agreement for DR grading and RNP measurements on the Optos imaging tool. By identifying factors that contribute to variability and proposing strategies to improve consistency, this research sought to contribute to the development of more reliable grading protocols.

## 2. Materials and Methods

This study was part of the INSPIRED-Q (Investigation of Retinal Non-Perfusion and Visual Function in Proliferative and Non-Proliferative Retinopathy in patients with Diabetes Quality of life). It was conducted at the National Institute for Health Research Moorfields Biomedical Research Centre, UK. The prospective part was approved by the National Research Ethics Committee (REC 24/LO/0669) on 17 September 2024. The retrospective part was approved by Moorfields Eye Hospital (Audit no. 1200) on 19 April 2023. The study adhered to the principles outlined in the Declaration of Helsinki. This present study retrospectively included patients with diabetic retinopathy (any stage) in at least one eye and those who had gradable Optos color fundus photos (CFP) and UWFA imaging in two separate visits. The exclusion criteria included poor-quality images or the presence of pan-retinal photocoagulation (PRP) during either of the two visits.

### 2.1. Image Acquisition

UWFA images were acquired on the Optos 200TX system (Optos Plc, Dunfermline, Scotland, UK) from patients diagnosed with diabetic retinopathy, according to the local Optos UWFA imaging protocol. The imaging protocol involved the intravenous administration of 5 mL of 10% fluorescein sodium, followed by image capture during the choroidal phase (up to 10 s), the arteriovenous phase (up to 15 s), the venous phase (up to 20 s), and the late phases (up to 10 min). A single investigator selected the optimal FFA image from the arteriovenous phase for each patient. The gamma value was adjusted and fixed to improve the visibility of peripheral lesions, microaneurysms, and non-perfusion areas, which was critical for accurate grading, and could vary from 1.0 to 2.0.

### 2.2. The INSPIRED Grid

**Figure 1:**
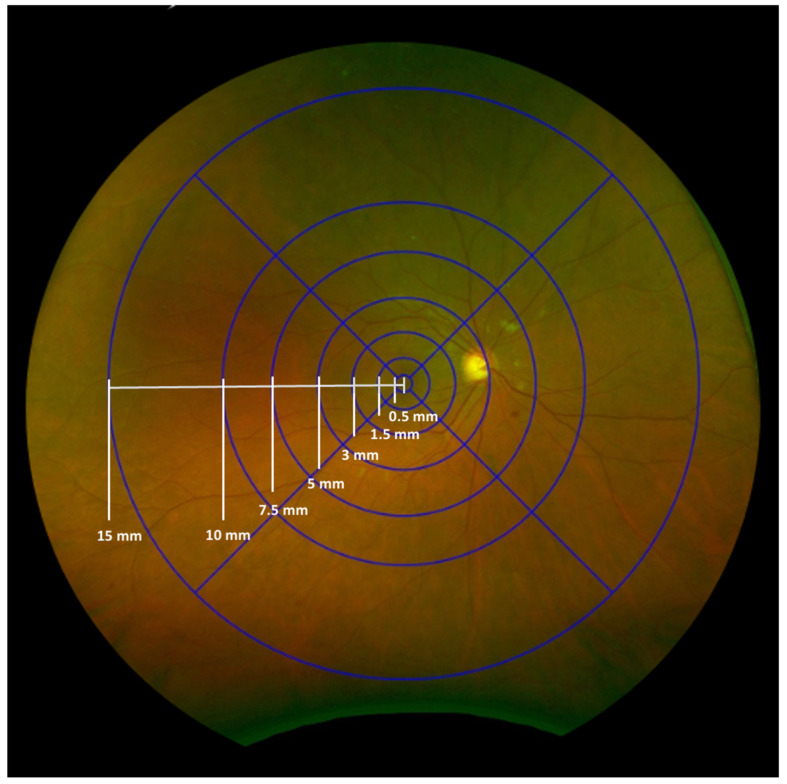
The INSPIRED-Q grid consists of seven fovea-centered concentric circles and four radial lines that divide the retina into zones and subfields, creating a total of 25 subfields (the most central circle counting as one subfield). The numbers on the grid indicate the radius of each circle.

Inspired by the ETDRS grid, we designed this “INSPIRED” grid using Optos’ proprietary tool. It consists of seven fovea-centered concentric circles and four radial lines dividing the retina into zones and subfields (Figure 1). The innermost circle, centered on the fovea, has a radius of 0.5 mm, defining the central subfield and covering approximately 0.785 mm^2^. Subsequent circles extend to radii of 1.5 mm (7.10 mm^2^), 3 mm (28.22 mm^2^), 5 mm (76.88 mm^2^), 7.5 mm (170.00 mm^2^), 10 mm (300.00 mm^2^), and 15 mm (620.00 mm^2^). The grid is divided into four quadrants (superior, inferior, nasal, and temporal) by two perpendicular lines that intersect at the fovea, creating a total of 25 subfields (with the central circle counting as one subfield). All measurements were performed using Optos’ proprietary tool, which applied trigonometry to adjust the area difference between the flattened image on the screen and the actual retinal areas in the eyes [19].

### 2.3. Evaluation of Ultrawide Field Color Images and Predominantly Peripheral Lesions

**Figure 2:**
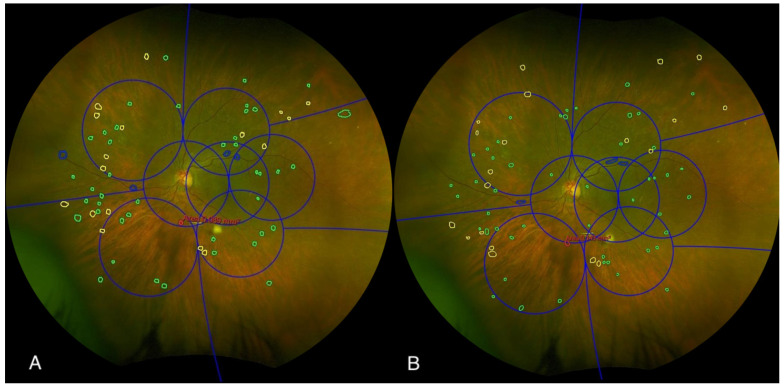
An example of two graders’ assessments of diabetic retinopathy characteristics (**A**,**B**) to determine whether the lesion is predominantly peripheral or predominantly central. Color fundus photograph of the left eye, graded independently by two masked graders using the Early Treatment Diabetic Retinopathy Study (ETDRS) grid (blue grid). The image illustrates the assessment of predominantly central lesions (PCL) within the ETDRS grid. Graders identified and delineated specific retinal lesions, including microaneurysms (marked with green circles), hemorrhages (marked with yellow circles), and venous beading (marked with blue circles). Additionally, intraretinal microvascular abnormalities (IRMA) were outlined in red, and the area of IRMA was quantified.

Ultra-widefield (UWF) color images were evaluated for DR severity using the International Clinical Diabetic Retinopathy (ICDR) grading system and the ETDRS Diabetic Retinopathy Severity Scale (DRSS) graded within the total gradable retina. The ICDR DR grading ranged from mild, moderate, or severe non-proliferative DR (NPDR) to PDR [20]. For each UWF image, the presence or absence of microaneurysms, hemorrhages, and venous beading was documented in each quadrant (superior, inferior, nasal, temporal). Intraretinal microvascular abnormalities (IRMA) and neovascularization elsewhere (NVE) were further characterized by their presence or absence and, if present, the area of involvement within each grid zone. The presence of neovascularization at the disc (NVD), preretinal hemorrhage (PRH), vitreous hemorrhage (VH), and fibrosis was also recorded for each quadrant (Figure 2). The presence of predominantly peripheral lesions (PPL) was assessed by determining whether more than 50% of DR lesions (e.g., hemorrhages, microaneurysms, venous beading, IRMA, and NVE) were located outside the standard ETDRS seven fields.

### 2.4. Evaluation of PPL and RNP

UWFA images were analyzed to quantify RNP and assess the distribution of PPL. Each eye was classified as either PPL-positive or PPL-negative based on the criterion mentioned above. Non-perfusion was defined as areas of capillary dropout or absence of fluorescein dye perfusion with brightness comparable to those of the foveal avascular zone (FAZ), excluding artifacts or areas obscured by media opacities. Using the Optos proprietary tool (with DICOM supplement), non-perfusion areas were manually delineated and recorded [21]. We did not annotate the RNP in the superior and inferior 10–15 mm sectors because of frequent eyelashes artifacts.

### 2.5. Intergrader Agreement

Qualitative and quantitative masked grading of UWFA images was performed by two pairs of medical retina fellows with at least 5 years of experience in medical retina. Our study included more than 25 images, which meets the minimum number of subjects required to apply a goodness-of-fit approach for assessing intergrader agreement [22]. The intergrader analysis was performed weekly, and a senior medical retina specialist discussed and adjudicated the discrepancies. Prior to independent grading, both fellows underwent standardized training, including a review of scoring criteria with a senior consultant and practice evaluations on a pilot image set (*n* = 10). Discrepant cases were identified by different gradings and were highlighted in Excel using conditional formulae. The flagged cases were resolved by a weekly hour-long joint discussion and adjudication by the senior medical retina consultant when necessary, and finally through blinded re-evaluation.

### 2.6. Statistical Analysis

The statistical analysis was performed to evaluate intergrader agreement for CFP and to quantify differences in RNP measurements. Intergrader agreement was assessed using Cohen’s kappa for qualitative grading and intraclass correlation coefficients (ICC) for quantitative RNP measurements. Cohen’s kappa measures the level of agreement between graders, accounting for agreement due to chance. A kappa value of 1 indicates perfect agreement, while a value of 0 indicates no agreement beyond chance. Bland–Altman analysis was used to evaluate systematic bias and variability between graders. All statistical analyses were performed using built-in functions and tools in Microsoft Excel 365.

## 3. Results

This study included a total of 100 comparisons from 50 eyes of 36 patients from two different visits. Demographic profile and percentages of DR severity, CFP PPL, and FFA PPL at baseline are reported in Table 1 and Table 2.

After eight consecutive weekly consensus meetings, the intergrader agreement for CFP improved from poor to excellent, with Cohen’s kappa values of 91% for ICDR severity, 89% for DRSS grading, and 89% for PPL. Final intergrader agreement was lowest in the moderate NPDR category (86%), while the highest agreement was observed for both mild NPDR and PDR (100%). For FFA PPL grading, excellent agreement was reached (kappa = 100%) (Table 3). One hundred comparisons were eligible for retinal non-perfusion assessment. After dividing the RNP by the gradable area, we compared the resultant NPI between two graders (Table 4). The total gradable area was 460.0 mm^2^/184.0 disc area (DA). The average non-perfusion area (NPA) was 96.3 mm^2^/38.5 DA with an average NPI of 0.21. The intraclass correlation coefficient (ICC) for total NPI was 0.49. Regional analysis revealed varying levels of agreement, with ICCs ranging from 0.40 to 0.57 across four different retinal sectors. The central region showed an ICC of 0.40, an NPI of 0.63, and an average NPA of 0.5 mm^2^/0.2 DA. The nasal region showed an ICC of 0.57, an NPI of 0.24, and an average NPA of 37.9 mm^2^/15.1 DA. The superior region showed an ICC of 0.44, an NPI of 0.17, and an average NPA of 12.8 mm^2^/5.1 DA. The temporal region showed an ICC of 0.45, an NPI of 0.21, and an average NPA of 32.9 mm^2^/13.1 DA. The inferior region showed an ICC of 0.40, an NPI of 0.16, and an average NPA of 12.2 mm^2^/4.9 DA.

**Figure 3:**
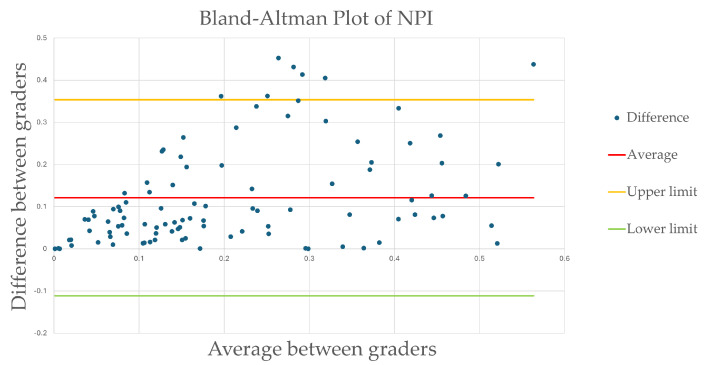
Bland–Altman plot of intergrader non-perfusion index. The mean NPI difference between the graders’ measurements was 0.12, with limits ranging from −0.11 to 0.35, and 7% of outliers (7/100) indicating an acceptable variability despite a few outliers.

Agreement between the two teams was further assessed using a Bland–Altman plot (Figure 3). The mean NPI difference between the graders’ measurements was 0.12, ranging from −0.11 to 0.35, and 7% of outliers (7/100), indicating an acceptable variability despite a few outliers.

## 4. Discussion

This study evaluated the intergrader agreement for qualitative and quantitative assessment of DR severity using UWF imaging, with a specific focus on PPL and RNP. The results demonstrate excellent intergrader agreement after training for qualitative grading of DR severity, PPL, and other DR-related features, as evidenced by high Cohen’s kappa values (98% for ICDR DR severity, 89% for DRSS grading, and 89% for PPL on CFP). Similarly, for UWFA, agreement for PPL grading reached 100%. The strong agreement observed in qualitative grading aligns with previous studies that have demonstrated the utility of UWF imaging in improving the accuracy and reproducibility of DR severity assessment [11,16,17,23,24]. These findings underscore the reliability of UWF imaging for qualitative DR assessment but also highlight the effectiveness of structured training and weekly consensus meetings in achieving high intergrader agreement. The high kappa values for PPL grading further emphasize the importance of incorporating peripheral retinal evaluation into DR assessment protocols, as PPLs have been shown to be associated with a higher risk of DR progression and more extensive non-perfusion [7,9,11,25].

However, while qualitative grading showed excellent agreement, quantitative assessment of RNP revealed moderate intergrader reliability, with an intraclass correlation coefficient (ICC) of 0.49 for the total gradable area. Regional analysis further demonstrated variability in agreement, with ICCs ranging from 0.40 to 0.57 across different retinal sectors. The nasal region showed the highest agreement (ICC = 0.57), while the inferior region exhibited the lowest (ICC = 0.40). These discrepancies may be attributed to challenges in visualizing and delineating non-perfused areas in certain retinal sectors, particularly in the periphery, where image quality and artifact interference can vary. The Bland–Altman analysis revealed a mean difference of 0.12 in the non-perfusion index (NPI) between graders, with limits of agreement ranging from −0.11 to 0.35, indicating acceptable variability but also highlighting the need for further standardization in RNP measurement protocols.

The variability in RNP measurements observed in this study is consistent with previous reports, which have highlighted the challenges of manual contouring and subjective interpretation of non-perfused areas [14,26,27,28,29,30]. Technical challenges in image acquisition and variable scan quality suggest the need for future implementations, such as standardized imaging protocols emphasizing uniform sector coverage, development of region-specific grading criteria accounting for anatomical variation in image quality, and incorporation of quality control checkpoints during acquisition to flag suboptimal scans for reacquisition or special annotation.

The time-consuming nature of manual RNP quantification and the potential for inter-observer variability underscore the need for automated or semi-automated tools to improve reproducibility. Recent advancements in artificial intelligence (AI) and machine learning offer promising solutions for automating the detection and quantification of non-perfusion, potentially reducing grader-dependent variability and improving efficiency [31,32]. However, the ground truth of images governs the development of AI algorithms. Future studies should explore the development, validation and then integration of AI-based algorithms into UWF imaging workflows to enhance the accuracy and reproducibility of RNP measurements.

The structured training program and weekly consensus meetings implemented in this study played a crucial role in achieving high intergrader agreement for qualitative grading. These findings underscore the importance of continuous training and calibration in enhancing the consistency of DR assessments, particularly in the context of complex imaging modalities, such as UWF. The success of this training approach suggests that similar programs could be implemented in clinical and research settings to enhance the reliability of DR grading and RNP quantification.

The implications of this study extend beyond the research setting, as the findings have important clinical applications. Accurate and reproducible assessment of DR severity and RNP is critical for guiding treatment decisions, particularly in the management of PDR and diabetic macular edema (DME) [27,28,33]. The ability to reliably identify and quantify PPLs using UWF imaging may improve risk stratification and enable more targeted interventions.

This study demonstrates significant methodological and clinical strengths. We used a standardized, ETDRS-inspired seven-circle grid (INSPIRED grid) to systematically evaluate retinal lesions and RNP quantification, introducing a critical advancement beyond traditional central-field grading. The excellent intergrader agreement (Cohen’s κ = 0.89–0.98) for DRSS and predominantly peripheral lesions (PPLs) underscores the reliability of ultra-widefield imaging (UWF) in real-world practice, while its 200° coverage enables detection of peripheral ischemia often missed by conventional methods. These findings validate UWF as a transformative tool for comprehensive DR management and highlight the need for standardized protocols to optimize reproducibility.

This study has several limitations. First, retrospective design and reliance on manual grading may introduce bias and variability. Second, the exclusion of images with poor peripheral quality or those that have undergone prior panretinal photocoagulation may limit the generalizability of the findings to more complex cases. Finally, the relatively small sample size of 50 eyes from 36 patients may restrict the statistical power of the analysis. Larger, prospective studies are needed to validate these findings and further refine grading protocols.

In conclusion, this study demonstrates good intergrader agreement for qualitative DR severity and PPL grading using UWF imaging, supporting its reliability for clinical use. However, agreement in RNP measurements was modest and highlights the need for improved standardization of ground-truth for potential integration of automated tools to enhance reproducibility. The findings underscore the importance of structured training and continuous calibration in achieving consistent and reliable DR assessments. Future research should focus on developing automated or semi-automated methods for non-perfusion quantification and PPL detection in real-world settings. By addressing these challenges, UWF imaging can play a pivotal role in advancing the management of diabetic retinopathy, ultimately reducing the risk of vision loss and improving patient outcomes.

## Figures and Tables

**Table 1:** Demographic profile at baseline.

Patient	*n* = 36
**Age**	54.2 (12.6)
**Male**	20 (56%)
**Ethnicity**	
Asian	7 (19%)
Black	1 (3%)
White	9 (25%)
Other	6 (17%)
Not stated	13 (36%)
**Bilateral recruitment**	14 (39%)

**Table 2:** Percentages of diabetic retinopathy (DR) severity, color fundus photographs (CFP) predominantly peripheral lesions (PPL) and fluorescein angiography (FFA) PPL at baseline.

Eyes	*n* = 50
**DR severity**	
Very mild NPDR (DRSS level 2)	1 (2%)
Mild NPDR (DRSS level 3)	6 (12%)
Moderate NPDR (DRSS level 4)	0 (0%)
Moderately severe NPDR (DRSS level 5)	4 (8%)
Severe NPDR (DRSS level 6)	22 (44%)
Mild PDR (DRSS level 7)	6 (12%)
Moderate PDR (DRSS level 8)	8 (16%)
High risk PDR (DRSS level 9)	3 (6%)
Very high-risk PDR (DRSS level 10)	0 (0%)
**CFP PPL presence**	9 (18%)
**FFA PPL presence**	11 (22%)

**Table 3:** Cohen’s kappa intergrader agreement on color fundus photographs (CFP) before and after training. Cohen’s kappa changed from 60% to 91% for the International Clinical Diabetic Retinopathy (ICDR) diabetic retinopathy (DR) severity, from 63% to 89% for the Diabetic Retinopathy Severity Scale (DRSS), from 69% to 89% for CFP predominantly peripheral lesions (PPL), and from 75% to 100% for fluorescein angiography (FFA) PPL. Cohen’s kappa intergrader agreement for ICDR categories changed from 90% to 100% for mild non-proliferative DR (NPDR), from 70% to 86% for moderate NPDR, from 60% to 88% for severe NPDR, and from 90% to 100% for proliferative DR (PDR).

	Before Training	After Training
**Numbers (patients; eyes; comparisons)**	14 patients; 21 eyes; 42 comparisons	36 patients; 50 eyes; 100 comparisons
**ICDR**	60%	91%
** Mild NPDR**	90%	100%
** Moderate NPDR**	70%	86%
** Severe NPDR**	60%	88%
** PDR**	90%	100%
**DRSS**	63%	89%
**CFP PPL**	69%	89%
**FFA PPL**	75%	100%

**Table 4:** Intergrader agreement on retinal non-perfusion index. ICC = intraclass correlation coefficient (ICC); NPI = non-perfusion index; DA = disc area.

FFA NPI from 36 Patients; 50 Eyes; 100 Comparisons				
	ICC	NPI	Average Area (mm^2^/DA)	Gradable Area (mm^2^/DA)
**Total**	0.49	0.21	96.3 mm^2^/38.5 DA	460.0 mm^2^/184.0 DA
**Centre**	0.40	0.63	0.5 mm^2^/0.2 DA	0.785 mm^2^/0.3 DA
**Nasal**	0.57	0.24	37.9 mm^2^/15.1 DA	154.8 mm^2^/61.9 DA
0.5–5 mm ring	0.06	0.04	0.7 mm^2^/0.3 DA	19.0 mm^2^/7.6 DA
5–10 mm ring	0.52	0.16	9.2 mm^2^/3.7 DA	55.8 mm^2^/22.3 DA
10–15 mm ring	0.60	0.35	28.0 mm^2^/11.2 DA	80.0 mm^2^/32.0 DA
**Superior**	0.44	0.17	12.8 mm^2^/5.1 DA	74.8 mm^2^/29.9 DA
0.5–5 mm ring	0.05	0.08	1.6 mm^2^/0.6 DA	19.0 mm^2^/7.6 DA
5–10 mm ring	0.51	0.20	11.2 mm^2^/4.5 DA	55.8 mm^2^/22.3 DA
**Temporal**	0.45	0.21	32.9 mm^2^/13.1 DA	154.8 mm^2^/61.9 DA
0.5–5 mm ring	0.41	0.11	2.1 mm^2^/0.8 DA	19.0 mm^2^/7.6 DA
5–10 mm ring	0.51	0.11	6.4 mm^2^/2.6 DA	55.8 mm^2^/22.3 DA
10–15 mm ring	0.46	0.31	24.5 mm^2^/9.8 DA	80.0 mm^2^/32.0 DA
**Inferior**	0.40	0.16	12.2 mm^2^/4.9 DA	74.8 mm^2^/29.9 DA
0.5–5 mm ring	0.31	0.08	1.5 mm^2^/0.6 DA	19.0 mm^2^/7.6 DA
5–10 mm ring	0.40	0.19	10.7 mm^2^/4.3 DA	55.8 mm^2^/22.3 DA

## Data Availability

Dr. Sivaprasad has full access to all the data in the study and takes responsibility for both the integrity of the data and the accuracy of the data analysis. The data will be made available upon request (sobha.sivaprasad@nhs.net).
